# Conditional Overexpression of Neuritin in Supporting Cell Protects Cochlear Hair Cell and Delays Age-Related Hearing Loss by Enhancing Autophagy

**DOI:** 10.3390/ijms26083709

**Published:** 2025-04-14

**Authors:** Shanshan Wang, Shaowei Lv, Junhao Hu, Yunfan Shi, Yu Li, Jianyun Zhang, Xiaohua Tan, Rong Chen, Yu Hong

**Affiliations:** School of Public Health, Hangzhou Normal University, Hangzhou 311121, China; wangshanshan@stu.hznu.edu.cn (S.W.); sw1010l@163.com (S.L.); hujunhao@stu.hznu.edu.cn (J.H.); shiyunfan@stu.hznu.edu.cn (Y.S.); liyu4832@163.com (Y.L.); 20180089@hznu.edu.cn (J.Z.); xiaohuatan@hznu.edu.cn (X.T.)

**Keywords:** neuritin, age-related hearing loss, autophagy, hair cells, LCB3, P21

## Abstract

Age-related hearing loss (ARHL) is a highly prevalent, burdensome sensorineural hearing loss closely associated with impaired autophagic influx. Our previous studies revealed that neuritin, a neurotrophic factor primarily expressed in the central nervous system, could alleviate drug-induced damages in hair cells (HCs) and spiral ganglion neurons. However, its effects on ARHL and whether these effects are closely related to autophagy remain unclear. Using the Nrn1 knock-in mice and cultured cochlear basilar membrane (CBM) of the neonatal mouse, we show that neuritin could restore aging-associated hearing loss and alleviate senescence-associated damage in the cochlea. Overexpression of neuritin in support cells (SCs) alleviates the loss of cochlear HCs and nerve fibers, reducing the damage to spiral ganglion neurons and the shifts in ABR’s high-frequency threshold. Furthermore, conditional overexpression of neuritin in SCs improves autophagic influx by upregulating the expression of microtubule-associated protein 1 light chain 3 type B (LCB3) protein and downregulating the expression of p21 protein. In cultured neonatal mouse CBM, neuritin administration significantly inhibits D-galactose-induced HC loss, cellular apoptosis, and ROS production and promotes autophagic influx. These effects were weakened when the autophagy inhibitor 3-MA was added. In summary, our results confirm the therapeutic potential of neuritin treatment for ARHL.

## 1. Introduction

Age-related hearing loss (ARHL), also known as senile deafness (presbycusis), is a progressive bilateral symmetric sensorineural hearing loss characterized by a high-frequency decline that occurs with increased age [1]. The pathogenesis of ARHL mainly includes loss of auditory hair cells, striae vascular degeneration, spiral ganglion apoptosis, and degeneration of the auditory nerve [2], with most of the cases having a mixture of these characteristics [3,4]. It was estimated that, globally, the number of prevalent cases and disability-adjusted life years (DALYs) of ARHL increased from 751.50 million and 22.01 million in 1990 to 1456.66 million and 40.24 million in 2019, respectively [5]. With the aging of the world’s population, the number of people with mild-to-complete hearing loss is projected to increase to 2.45 billion (95% uncertainty interval (UI): 2.35 billion –2.56 billion) by 2050 [6]. ARHL could seriously impede spoken communication in older individuals [2] and also lead to a lot of other adverse health outcomes, such as higher medical costs [7], cognitive decline [8], Alzheimer’s diseases [9], falls and frailty [10], and even death [11]. Though ARHL is believed to be a preventive disease, and multiple pharmacologic or genetic therapies have been used to reduce the progression of age-related hearing loss or to restore hearing, efforts have been largely unsuccessful to date [12].

Defining the underlying mechanisms of ARHL is the first step toward targeted interventions. Though the molecular mechanisms underlying ARHL have not been fully elucidated, age-related reactive oxygen species (ROS) accumulation and mitochondrial dysfunction, fibrocyte regeneration, stem cell depletion, senescence-associated inflammation, and impaired autophagy are proposed to be some of the most critical mechanisms engaged in the pathogenesis of this condition [13,14,15]. Continuous oxidative stress could accelerate cellular aging and induce hearing loss in a short-term period [16]. The dysregulation of mitochondrial dynamics, including mitochondrial fission and pyroptosis, exacerbated the aging process of hair cells [17]. Aberrant autophagy, which makes the clearance of the misfolding and aggregated proteins and organelles ineffectively, could also promote cochlear cell death and significantly contribute to the development of ARHL [14]. Results from preclinical studies showed that treatments that inhibit ROS production [18,19], regulate mitochondrial dysfunction [20], and enhance autophagy [21,22] could prevent or delay the progression of ARHL. Substances that exert multiple regulatory actions on these pathways have positive and encouraging results [23,24]. Despite these findings, clinical research has not made breakthrough progress, and the data from an RCT showed that the administration of antioxidants did not affect any of the evaluated measures of hearing [25]. Therefore, additional efforts are warranted to develop novel treatment strategies for ARHL.

Evidence from different studies showed that neurotrophic factors may also have protective effects on ARHL [26]. Neuritin (candidate plasticity-related genes 15, CPG15/NRN1), also known as neuregulin, is a neurotropic factor vital in nervous system development and plasticity [27]. It is anticipated to play a pivotal role in the functional recovery of injured central nerves, encompassing intracerebral hemorrhage [28], spinal cord damage [29], and brain ischemia–reperfusion injury [30]. Moreover, results from our lab found that neuritin had protective effects against drug-induced hearing loss (ototoxic hearing loss) [31,32]. Our previous studies found that conditional overexpression of neuritin in the cochlear mitigated drug-induced hair cell (HC) damage and directly induced HC regeneration from support cells (SCs) [32] in mice. It could also restore ouabain-induced hearing loss in gerbils by maintaining the number and arrangement of spiral ganglion neurons (SGNs) and nerve fibers in the damaged cochlea [31]. As the loss of HCs and SGNs are the typical pathological features shared between drug-induced hearing loss and ARHL [33], it was worth investigating the potential benefits of neuritin on ARHL. In addition, neuritin was demonstrated to have pronounced effects on autophagic flux in neuron cells [34,35], a critical mechanism underlying ARHL [36]. Neuritin is a downstream target of neurotrophin-3 (NT-3) [37], a neurotrophic factor that has potential protective effects against ARHL [38]. These results coherently illustrate the potential benefits of neuritin in managing ARHL.

In this study, using a mouse model of ARHL, we evaluate the protective effects of neuritin on ARHL and explore the underlying mechanisms, especially the autophagy signaling pathways. We find that conditional overexpression of neuritin in cochlear support cells (SCs) could restore auditory hair cells and protect against damage in cochlear hair cells (HCs), spiral ganglion neurons (SGNs), and nerve fibers caused by aging. Mechanistically, we find that neuritin overexpression promotes autophagic flux activation in the cochlea. These observations are confirmed by the in vitro study that finds that, in cultured neonatal mouse cochlear basilar membrane (CBM), neuritin administration significantly inhibits d-gal-induced senescence, cellular apoptosis, and ROS production in the CBMs via promoting autophagic influx. These effects are weakened when the autophagy inhibitor 3-methyladenine (3-MA) is added. Overall, these findings demonstrate the therapeutic potential of neuritin on ARHL and may help develop a new approach for the prevention and treatment of ARHL.

## 2. Results

### 2.1. The Expression of Neuritin in the Cochlea of Wild-Type and Neuritin Knock-In Mice

**Figure 1:**
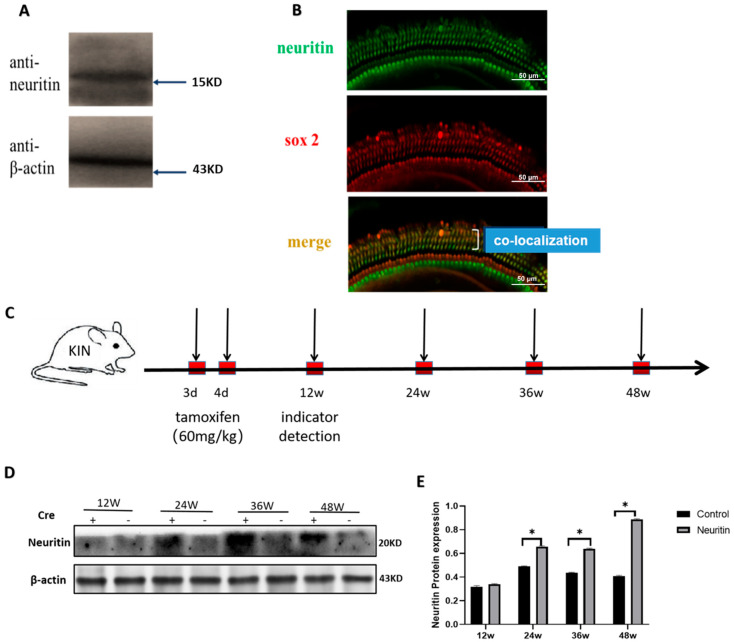
The cellular localization and conditional overexpression of neuritin in the cochlea of mice. (**A**,**B**) The expression levels and cellular location of neuritin protein in the wild-type mouse cochlea (5-week-old). Green represents anti-EGFP labeling neuritin; red represents anti-Sox2 labeling supporting cells (SCs) (scale bar = 50 µm). (**C**) Schematic timeline of conditional overexpression and detection time of neuritin protein in Sox2^CreER/+^Neuritin^stop/stop^ mouse (neuritin). (**D**,**E**) Western blots and quantitative analysis of neuritin expression levels in the cochlear of Nrn1^stop^ mouse (control) and Sox2^CreER/+^Neuritin^stop/stop^ mouse (neuritin) at different time points. Statistical analyses were performed by Student’s *t*-test; *, *p* < 0.05.

Western blot and immunofluorescence co-localization analyses confirmed that neuritin could be detected in the cochlea of wild-type mice (5-week-old), and it was primarily localized within supporting cell bodies (Figure 1A,B). Conditional knockout of the neuritin gene in cochlear supporting cells (SCs) resulted in a reduced expression of neuritin protein and a significant elevation in hearing threshold elicited by click auditory brainstem response (ABR) (Figure S1). On the other hand, conditional overexpression of neuritin gene in mice (Sox2^CreER/+^ Nrn1^stop (+/-)^ mice) exhibited significantly higher levels of neuritin protein than those of control mice (Nrn1^stop/stop^ mice) at the time points of 24, 36, and 48 weeks (Figure 1C–E).

### 2.2. Conditional Overexpression of Neuritin Protects Against Damages in Cochlear Hair Cells, Nerve Fibers, and Spiral Ganglion Neurons (SGNs) Caused by Aging

**Figure 2:**
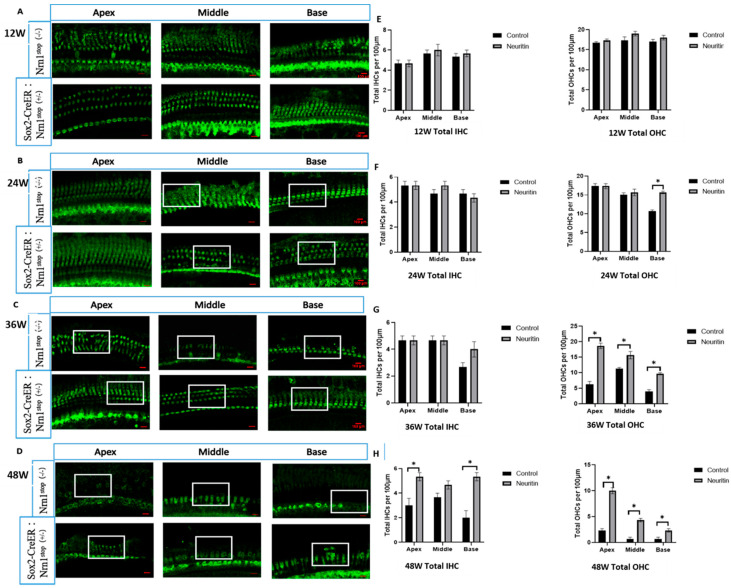
Conditional overexpression of neuritin in supporting cells (SCs) ameliorates aging-induced hair cell (HC) damage in the cochlear. (**A**–**D**) The HCs in the apex, middle, and base loops of the cochlear basilar membrane in Sox2^CreER/+^ Nrn1^stop (+/-)^ mice (neuritin) and Nrn1^stop (-/-)^ mice (control) at the time points of 12, 24, 36, and 48 weeks; the white rectangles show the typical zones of HCs in the basal, middle, and apical regions of the cochlear basilar membrane. Green represents anti-Myo7A labeling HCs (scale bar = 100 μm). (**E**–**H**) Quantity analysis of HCs in the apex, middle, and base loops of the cochlear basement membrane in mice with different ages. IHC, inner hair cell; OHC, outer hair cell. Statistical analyses were performed by Student’s *t*-test; *, *p* < 0.05.

To evaluate the protective effects of neuritin on ARHL, we established a model of ARHL with Sox2^CreER/+^Neuritin^stop/stop^ C57BL/6J mice (neuritin, experimental group) and Nrn1^stop^ C57BL/6J mice (Nrn1stop, control group) with a total observation period of 48 weeks. The differences in the number of HCs, density of nerve fibers, and spiral ganglion neurons in the cochlear epithelium of mice were evaluated and compared between the two groups. The results showed that from the time points of 24 weeks to 48 weeks, there was a heavy loss of HCs at the base of the cochlea in the mice of the control group (Figure 2). These losses were also observed at the middle and apex of the cochlear epithelium in the mice of the control group at the time points of 36 and 48 weeks. Compared with the mice of the control group, HCs were significantly preserved in the mice of the experimental group at different time points (24, 36, and 48 weeks) (*p* < 0.05) (Figure 2).

**Figure 3:**
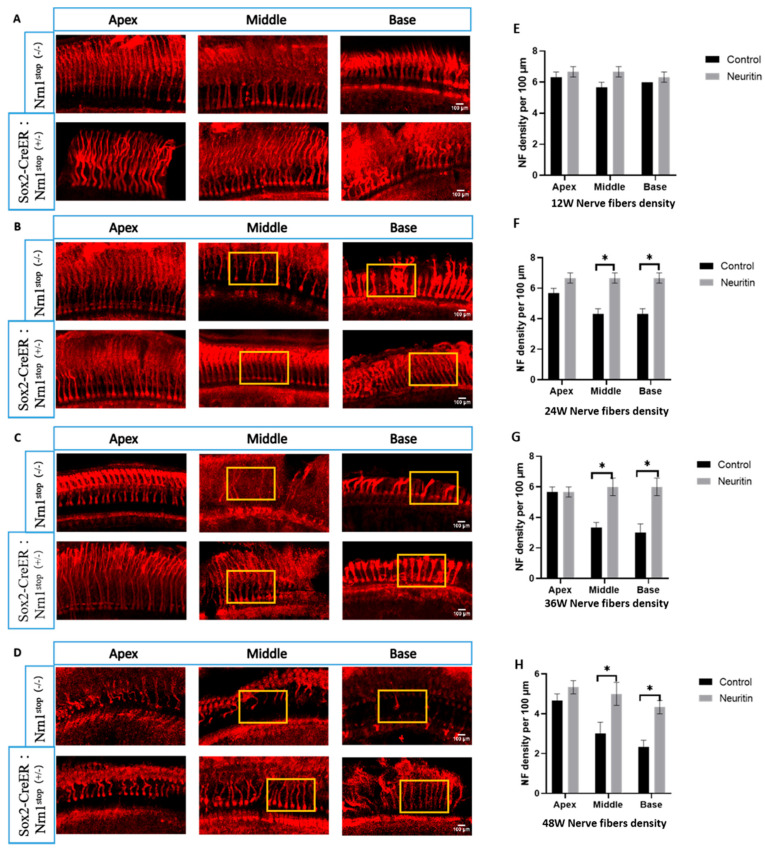
Conditional overexpression of neuritin in supporting cells (SCs) reduces aging-induced nerve fiber damages in the cochlear. (**A**–**D**) The nerve fibers in the apex, middle, and base loops of the cochlear basilar membrane in Sox2^CreER/+^ Nrn1^stop (+/-)^ mice (neuritin) and Nrn1^stop (-/-)^ mice (control) at the time points of 12, 24, 36, and 48 weeks. Red represents NF200-positive nerve fibers; scale bar = 100 μm. Yellow rectangles show the typical zone of nerve fibers in the apex, middle, and base loops of the cochlear basilar membrane. (**E**–**H**) Quantification of nerve fibers in the apex, middle, and base loops of cochlear basement membrane in mice at different time points (*n* = 6 for each group). Statistical analyses were performed by Student’s *t*-test; *, *p* < 0.05.

We assessed the effects of neuritin overexpression in SCs on the aging-induced devoid of nerve fibers within the cochlea by fluorescence immunohistopathologic analysis. The results showed that mice in the neuritin group had a significantly higher density of nerve fibers in the cochlea than those in the control group. From 24 weeks to 48 weeks, in the middle and base of the cochlear epithelium in mice, a significantly higher density of nerve fibers was observed in the middle and base of the cochlear epithelium of mice in the neuritin group than those of the control group (Figure 3).

**Figure 4:**
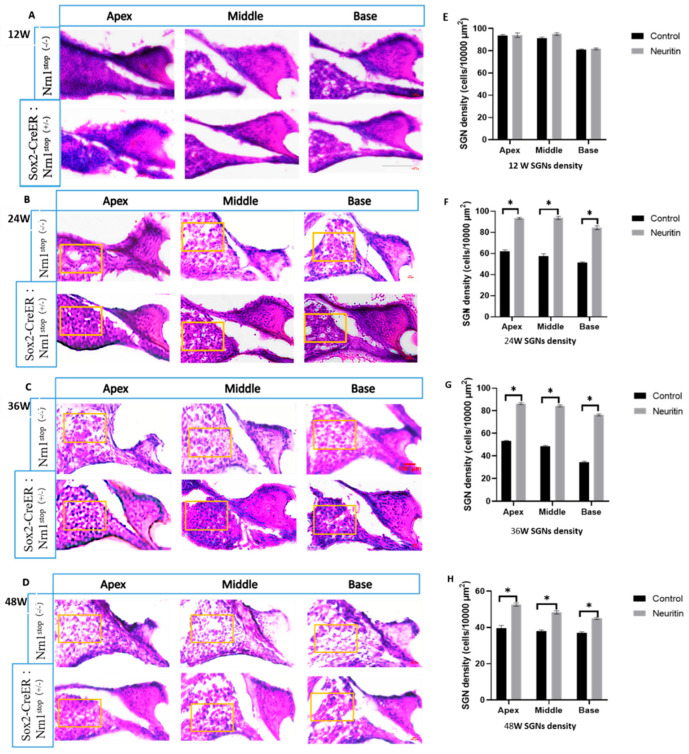
Conditional overexpression of neuritin in supporting cells (SCs) reduces aging-induced spiral ganglion neuron (SGN) damage in the cochlear. (**A**–**D**) HE staining results of spiral ganglion neurons in the apex, middle, and base of the cochlear basilar membrane in Sox2^CreER/+^ Nrn1^stop (+/-)^ mice (neuritin) and Nrn1^stop (-/-)^ mice (control) at the time points of 12, 24, 36, and 48 weeks observed by H&E staining; scale bar = 100 μm. Yellow rectangles show the typical zone of SGNs in the cochlear basilar membrane’s apex, middle, and base loops. (**E**–**H**) Quantification of SGN density in the apex, middle, and base loops of the cochlear basement membrane in mice with different ages (*n* = 6 for each group). Statistical analyses were performed by Student’s *t*-test; *, *p* < 0.05.

Moreover, the density of spiral ganglion neurons (SGNs) in the cochlea of the mice between the neuritin and control groups was compared to further confirm the effects of neuritin overexpression on ARHL. Consistent with the observations in HCs and nerve fibers, neuritin overexpression in SCs significantly preserved aging-induced SGN loss in the apex, middle, and base of the cochlear of the mice at the different time points (24, 36, and 48 weeks) (*p* < 0.05) (Figure 4).

### 2.3. Conditional Overexpression of Neuritin Restores Aging-Associated Hearing Loss and Enhances Autophagic Influx in the Cochlea of the Mice

**Figure 5:**
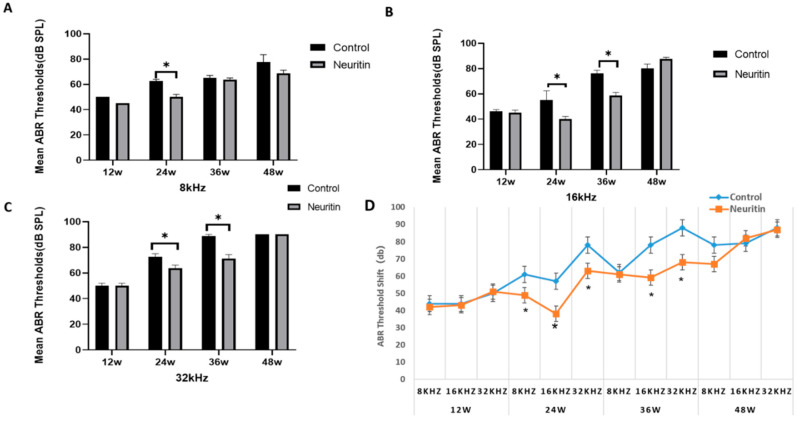
Neuritin overexpression rescues high-frequency hearing threshold shifts in mice with age-related hearing loss. (**A**–**C**) The hearing thresholds elicited by click or high-frequency (8000 Hz, 16,000 Hz, and 32,000 Hz) stimuli in Sox2^CreER/+^ Nrn1^stop (+/-)^ mice (neuritin) and Nrn1^stop (-/-)^ mice (control) at the time points of 12, 24, 36, and 48 weeks. (**D**) Line chart of ABR hearing threshold shifts at different frequencies in Sox2 ^CreER/+^ Nrn1^stop (+/-)^ mice (neuritin) and Nrn1stop ^(-/-)^ mice (control) at the time points of 12, 24, 36, and 48 weeks. Statistical analyses were performed by Student’s *t*-test; *, *p* < 0.05.

The results from the auditory brainstem response (ABR) test indicated that aging resulted in gradual increments in hearing thresholds elicited by click or high-frequency (8000 Hz, 16,000 Hz, and 32,000 Hz) stimuli in the mice at the time points of 12 weeks to 48 weeks (Figure 5). Compared with Nrn1^stop/stop^ mice (control), Sox2^CreER/+^Neuritin^stop/stop^ mice exhibited a significantly lower hearing threshold at the time point of 24 weeks elicited by 8000 Hz, 16,000 Hz, and 32,000 Hz stimuli. They had a considerably lower hearing threshold elicited by 16,000 Hz and 32,000 Hz stimuli at 36 weeks. However, no significant differences in hearing threshold were observed between the experimental and control group mice at 48 weeks (Figure 5).

**Figure 6:**
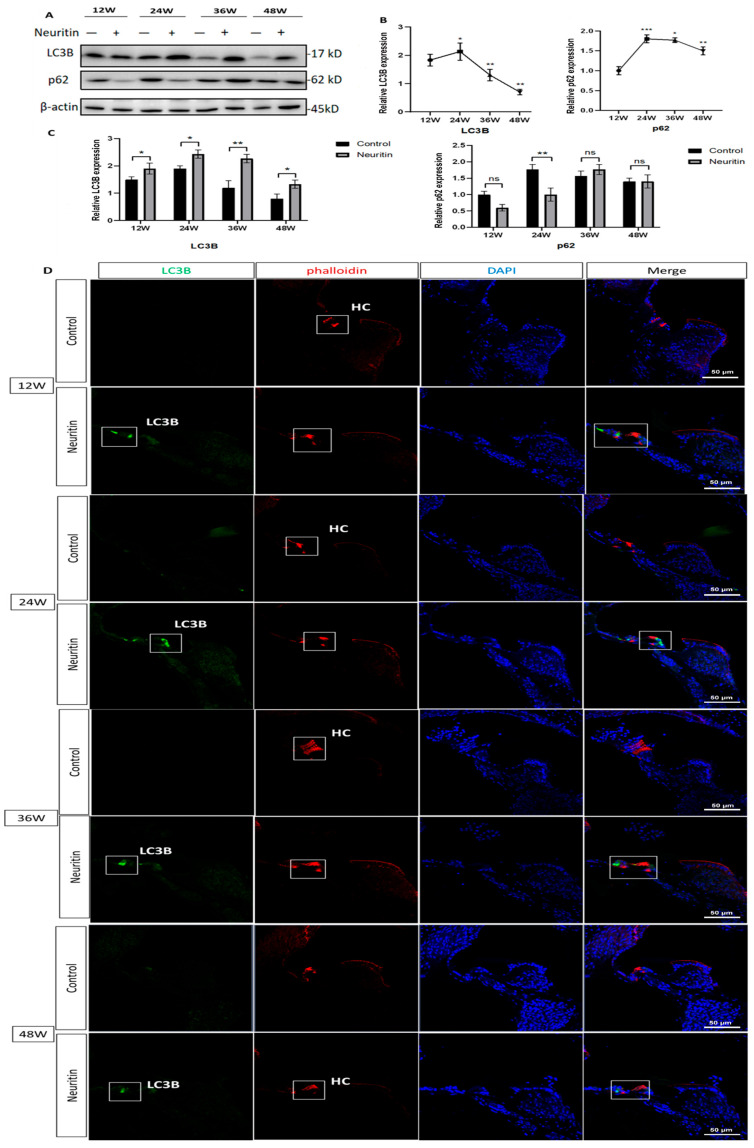
Conditional overexpression of neuritin in supporting cells (SCs) improves autophagic influx in the cochlea in mice with age-related hearing loss. (**A**,**B**) The changes in LC3B and p62/SQSTM1 protein expression levels in the cochlear of Sox2^CreER/+^ Nrn1^stop (+/-)^ mice (neuritin) and Nrn1^stop (-/-)^ mice (control) at different time points (12, 24, 36, and 48 weeks) detected by Western blot analysis. (**C**) Quantitative analysis of LC3B and p62/SQSTM1 protein expression levels in the cochlear of Sox2^CreER/+^ Nrn1^stop (+/-)^ mice (neuritin) and Nrn1^stop (-/-)^ mice (control) (*n* = 6 per group). Statistical analyses were performed using Student’s *t*-test. *, *p* < 0.05; **, *p* < 0.01; ***, *p* < 0.001. (**D**) Fluorescence immunohistopathologic evaluation of the localization and expression levels of LC3B protein in cochlea of Sox2^CreER/+^ Nrn1^stop (+/-)^ mice (neuritin) and Nrn1^stop (-/-)^ mice (control). Green represents fluorescently labeled LC3B protein; red represents phalloidin-stained hair cells (HCs); blue is DAPI staining of cell nuclei and white rectangles represent phalloidin staining hair cells. DAPI, 4′,6-diamidino-2-phenylindole. Scale bar = 50 μm. Statistical analyses were performed by Student’s *t*-test; *, *p* < 0.05; **, *p* < 0.01; ***, *p* < 0.001.

The expression levels of p62 and LC3B proteins in the cochlea of the mice were detected to evaluate the effects of neuritin on the autophagic influx. The results showed that the expression levels of LC3B and p62 proteins in the cochlea of the mice significantly increased from 12 week to 24 weeks and then decreased at the time points of 36 weeks and 48 weeks (Figure 6A,B). Compared with Nrn1^stop/stop^ mice (controls), Sox2^CreER/+^Neuritin^stop/stop^ mice expressed significantly higher levels of LC3B in the cochlea at the time points of 12, 24, 36, and 48 weeks (Figure 6C). On the contrary, Sox2^CreER/+^Neuritin^stop/stop^ mice expressed significantly lower levels of p62 in the cochlea compared with those of Nrn1st^op/stop^ mice at the time point of 24 weeks (*p* < 0.05). There were no significant differences in the expression levels of p62 protein between mice of the experimental and control groups at the time points of 36 and 48 weeks (*p* > 0.05) (Figure 6C). The results from immunofluorescence staining also found that Sox2^CreER/+^Neuritin^stop/stop^ mice expressed significantly higher levels of LC3B in the cochlea compared with the Nrn1st^op/stop^ mice at the time points of 12, 24, 36, and 48 weeks (Figure 6D).

### 2.4. D-Gal Induced Senescence in Cultured Mouse Cochlear Basilar Membranes (CBMs)

**Figure 7:**
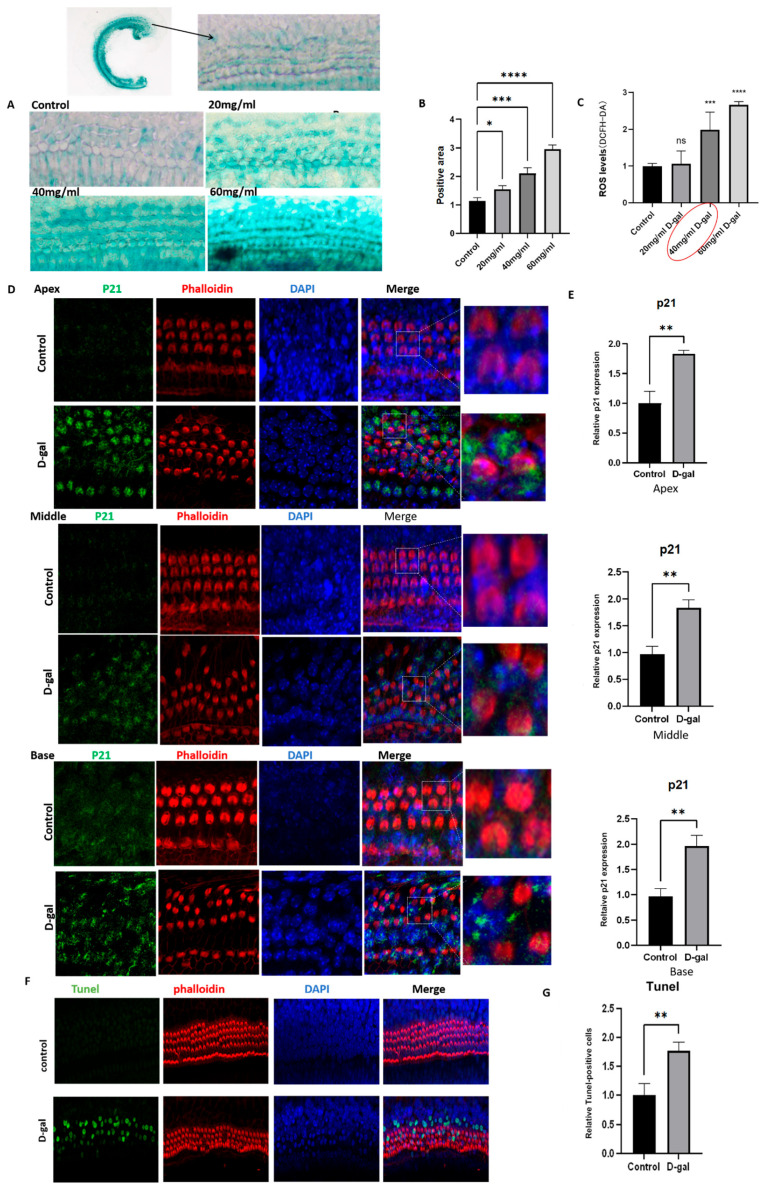
Establishment and characterization of senescence-associated damages in the cultured neonatal mouse cochlear basilar membranes (CBMs). (**A**,**B**) Senescence-associated D-galactosidase (SA-D-gal) staining and the relative levels of D-gal-positive staining cells in cultured CBMs of neonatal mice treated with different dosages of D-gal (20 mg/mL, 40 mg/mL, and 60 mg/mL) (*n* = 6 for each group). Scale bar = 50 μm. Statistical analyses were performed by one-way ANOVA followed by the Bonferroni post hoc test. *, *p* < 0.05; **, *p*< 0.01; ***, *p* < 0.001; ****, *p* < 0.0001. (**C**) The relative reactive oxidative species (ROS) levels detected by DCFH-DA in cultured CBMs of neonatal mice treated with different dosages of D-gal (20 mg/mL, 40 mg/mL, and 60 mg/mL) (*n* = 6 for each group). The red border implies the dose of D-gal (40 mg/mL) used in the subsequent experiments. (**D**,**E**) Fluorescence immunostaining and quantitative analysis of p21 protein expression levels in the apex, middle, and base of cultured CBMs in D-gal-treated (40 mg/mL) and control groups (*n* = 6 per group). Scale bar = 20 μm. Green represents fluorescently labeled p21 protein; red represents phalloidin-stained hair cells (HCs); purple represents DAPI-stained nucleus. The dotted rectangle represents the enlarged area of the cross-section for each condition. (**F**,**G**) Fluorescence immunohistopathologic evaluation and quantitative analysis of the expression level of TUNEL-positive cells (green) in D-gal-treated (40 mg/mL) and control groups (*n* = 6 for each group); scale bar = 50 μm. DAPI, 4′,6-diamidino-2-phenylindole.

In this study, different doses of d-galactose (d-gal) (20 mg/mL, 40 mg/mL, and 60 mg/mL) were used to induce cultured mouse cochlear basilar membrane (CBM) senescence to establish a model of ARHL in vitro (Figure 7A–C). The results showed that 40 mg/mL d-gal treatment resulted in significant and sustained increments in the area of SA-β-gal-positive staining cells and ROS levels in the cultured mouse CBMs compared with those of the control group. Thus, a dose of 40 mg/mL d-galactose was used in the subsequent experiments. The results from fluorescence immunostaining showed that d-gal treatment significantly increased the expression levels of p21 protein in the apex, middle, and base loops of cultured CBMs compared with those in the control group (Figure 7D,E). Moreover, the d-gal treatment also resulted in a significantly higher level of cellular apoptosis in the cultured CBMs compared with that of the control group (Figure 7F,G).

### 2.5. Neuritin Administration Mitigates d-Galactose-Induced Senescence, ROS Production, and Cellular Apoptosis, Enhancing Autophagic Influx in Cultured Neonatal Mouse Cochlear Basilar Membranes (CBMs)

**Figure 8:**
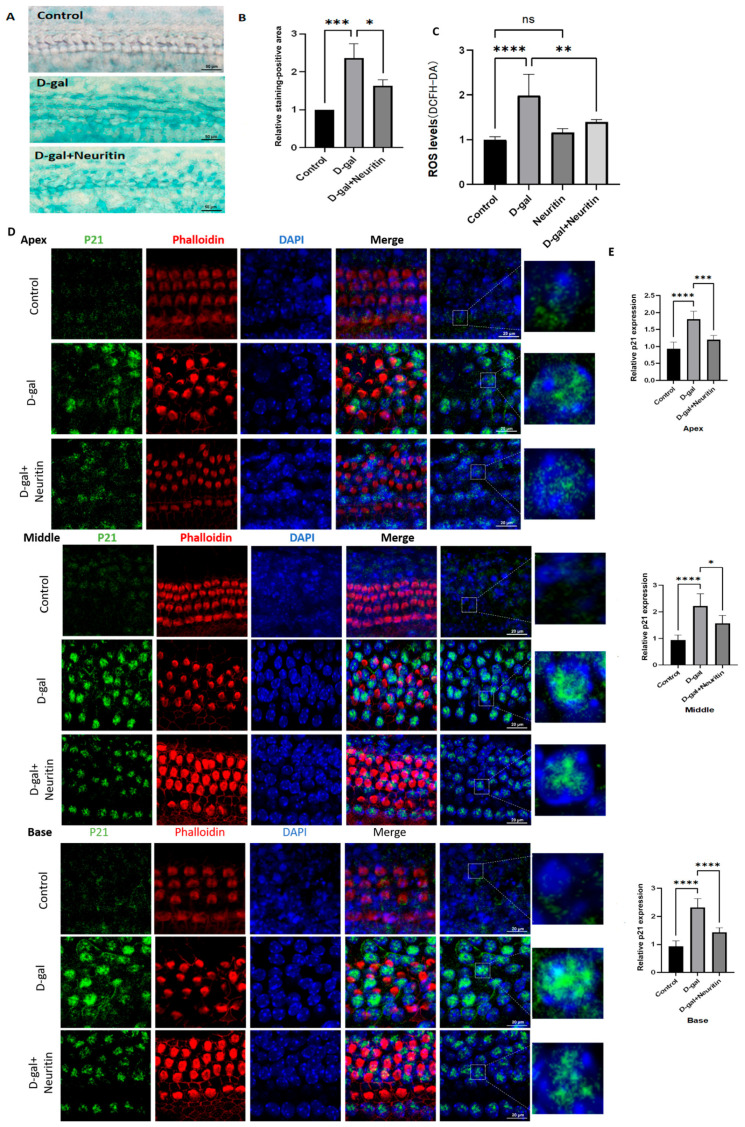
Neuritin ameliorates d-galactose-induced senescence damage in the cultured neonatal mouse cochlear basilar membranes (CBMs). (**A**) Representative images of SA-β-Gal staining (blue) in the cultured neonatal mouse cochlear basilar membranes (CBMs) from different groups (control, 40 mg/mL D-gal, and 40 mg/mL D-gal + 16 µg/mL neuritin). Scale bar = 50 μm. (**B**) The relative levels of SA-β-gal-positive staining cells in the different groups. Data are shown as mean ± SD (*n* = 6 in each group). (**C**,**D**) Fluorescence immunohistopathologic evaluation and quantitative analysis of the expression levels of P21 protein in different groups (*n* = 6 in each group); scale bar = 20 μm. Green represents fluorescently labeled p21 protein; red represents phalloidin-stained hair cells (HCs); purple represents DAPI-stained nucleus. (**E**) ROS levels detected by DCFH-DA in different groups (*n* = 6 per group). The dotted rectangle represents the enlarged area of the cross-section for each condition. Statistical analyses were performed by one-way ANOVA followed by LSD post hoc test. *, *p* < 0.05; **, *p*< 0.01; ***, *p* < 0.001; ****, *p* < 0.0001; ns, no statistical significance. DAPI, 4′,6-diamidino-2-phenylindole.

To evaluate the effects of neuritin on the d-gal-induced senescence-associated damage in cultured neonatal mouse CBMs, a dose of 16 µg/mL neuritin was used in this study. The results showed that compared with the D-gal group, the neuritin+D-gal group had a significantly lower area of SA-β-gal-positive staining cells (Figure 8A,B), ROS level (Figure 8C), and the p21 protein expression levels in the apex, middle, and base loops of cultured CBMs (Figure 8D,E).

**Figure 9:**
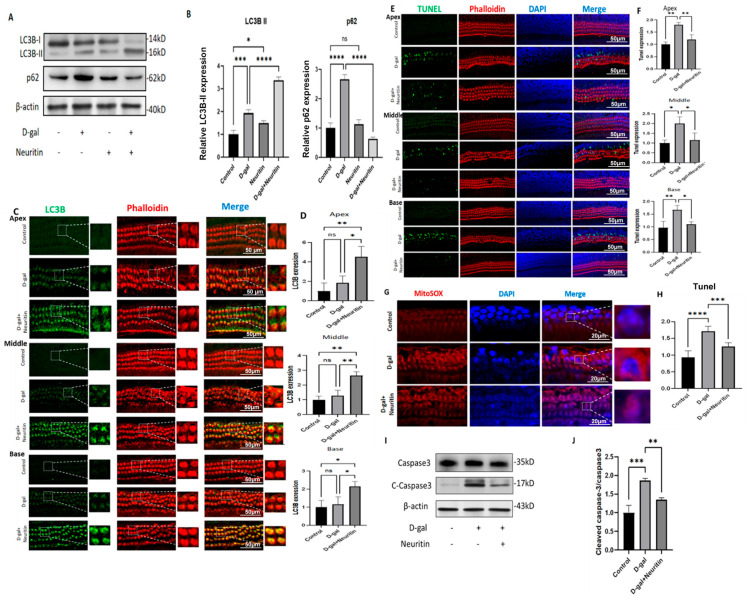
Neuritin ameliorates d-gal-induced damages in cultured neonatal mouse cochlear basilar membranes (CBMs) by enhancing autophagic influx. (**A**,**B**) Western blot and quantitative analysis of the expression levels of LC3BI/II, p62/SQSTM1 proteins in the CBMs of different groups (control, d-gal, neuritin, and neuritin +d-gal) (*n* = 6 per group). (**C**,**D**) Fluorescence immunohistopathologic evaluation and quantitative analysis of the expression level of LC3B protein in the CBMs of different groups; scale bar = 50 μm. Green represents fluorescently labeled LC3B protein, and red represents phalloidin-stained hair cells (HCs). (**E**,**F**) Fluorescence immunohistopathologic evaluation and quantitative analysis of the expression levels of TUNEL-positive cells in the CBMs in different groups; scale bar = 50 μm. Green represents fluorescently labeled TUNEL-positive cells. (**G**,**H**) Fluorescence immunohistopathologic evaluation and quantitative analysis of mitochondrial reactive oxidative species (ROS) levels evaluated by MitoSOX red staining in different groups. Red represents MitoSOX-stained mitochondria in hair cells (HCs); purple represents DAPI-stained nucleus. Scale bar = 20 μm. The dotted rectangle represents the enlarged area of the cross-section for each condition. (**I**,**J**) Western blot and quantitative analysis of the expression level of caspase3 and C-caspase3 proteins in the CBMs of different groups (*n* = 6 in each group). Statistical analyses were performed by one-way ANOVA followed by LSD post hoc test. *, *p* < 0.05; **, *p*< 0.01; ***, *p* < 0.001; ****, *p* < 0.0001; ns, no statistical significance.

Moreover, consistent with *the* in vivo study, neuritin administration significantly increased the expression levels of LC3B protein. It decreased the expression levels of p62 protein (Figure 9A–D) in the apex, middle, and base loops, inhibiting d-galactose-induced ROS production (Figure 9G,H) and cellular apoptosis (expression of caspase-3) (Figure 9E,F) in cultured neonatal mouse CBMs.

### 2.6. The Protective Effects of Neuritin on d-Galactose-Induced Senescence Damages Were Weakened by Administration of Autophagy Inhibitor 3-MA

**Figure 10:**
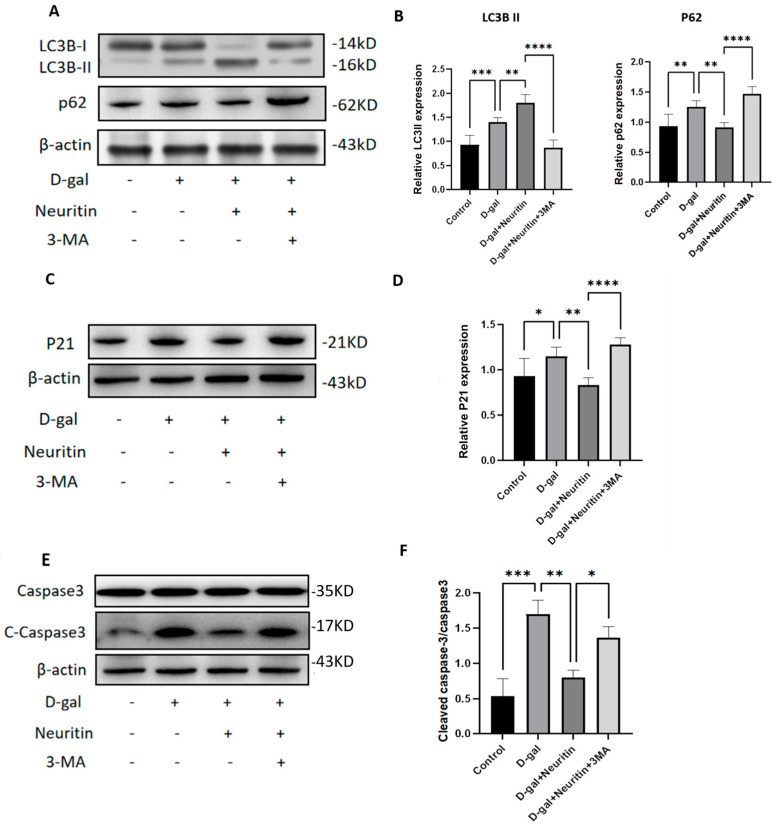
The protective effects of neuritin against d-gal-induced damages in cultured neonatal mouse cochlear basilar membranes (CBMs) were weakened by the administration of autophagy inhibitor 3-MA. Western blot and quantitative analysis of the expression level of LC3BI/II, p62/SQSTM1 (**A**,**B**), p21 (**C**,**D**), and caspase3 and C-caspase3 proteins (**E**,**F**) in the cultured neonatal mouse cochlear basilar membranes (CBMs) receiving different treatments (control, 40 mg/mL D-gal, 40 mg/mL D-gal + 16 μg/mL neuritin, and 40 mg/mL D-gal + 16 μg/mL neuritin + 2 nmol/mL 3-MA; *n* = 6 per group). Statistical analyses were performed by one-way ANOVA followed by LSD post hoc test. *, *p* < 0.05; **, *p*< 0.01; ***, *p* < 0.001; ****, *p* < 0.0001.

We proceeded to determine the role of autophagy in the protective effects of neuritin on d-gal-induced senescence damages in cultured CBMs using autophagy inhibitor 3-MA. The results showed that, compared with the D-gal group, D-gal +Neuritin treatment could significantly improve d-gal-induced reduction in LC3B protein expression but significantly inhibit d-gal-induced increase in the expression of p21 and p62 proteins and c-caspase-3/caspase-3 ratio (Figure 10). However, all these changes were reversed by 3-MA co-administration. Compared with the D-gal +Neuritin group, the D-gal +Neuritin+3-MA group had a significantly lower expression level of LC3B protein but had significantly higher expression levels of p62 and p21 proteins and higher c-caspase-3/caspase-3 ratio (Figure 10).

## 3. Discussion

As ARHL is a complex condition involving various pathological mechanisms, treatments with multiple actions on these mechanisms may have more pronounced effects. In the present study, we unveil the role of neuritin in preventing aging-related damages in HCs, nerve fibers, and spiral ganglia, restoring auditory function, and improving autophagic influx in vivo. We further demonstrate that neuritin can inhibit cellular senescence, ROS production, and apoptosis, enhancing autophagic influx in vitro. These results suggest that neuritin administration can substantially improve ARHL through diverse pathways.

In our study, conditional overexpression of neuritin in SCs not only ameliorates hair cell damage but preserves nerve fibers and spiral ganglion neurons (SGNs) in the mouse model of ARHL. Mechanistically, we demonstrated that neuritin administration could reduce senescence-associated oxidative stress and cellular apoptosis. These results indicated that neuritin had both protective effects on sensory presbycusis (characterized by the loss of sensory hair cells) and neural presbycusis (characterized by declines in the number of spiral ganglion cells) induced by aging [39]. These outcomes were consistent with our earlier observations, which showed that neuritin could effectively maintain the arrangement of nerve fibers after rat sciatic nerve injury [40] and could restore the number and arrangement of SGNs and nerve fibers in gerbils after drug-induced cochlear damage [31]. Though the mechanisms underlying these effects remain to be unveiled, the multiple impacts of neuritin in the nervous system, including neuritogenesis, neurite arborization, and neurite extension, suggest that it is a promising candidate [41]. Moreover, previous studies showed that neuritin could enhance the regeneration of HCs after injury and HCs could support spiral ganglion neuron survival by producing several peptide neurotrophic factors such as neurotrophin-3 and glial-derived neurotrophic factor [42]. Therefore, neuritin may indirectly protect SGNs from aging-induced damage by preserving HCs in the cochlear. This was supported by the finding that neurotrophin-3 could increase the synapse density between SGNs and HCs, which had a protective effect on hidden hearing loss [43]. Hidden hearing loss is a condition characterized by a combination of hearing difficulties in the absence of clinically abnormal audiometric thresholds, which could be a pre-stage of both ARHL and noise-induced hearing loss [44,45]. Thus, this result illustrated the potential preventive value of neuritin for hearing loss. Consistent with previous studies [35,46,47], neuritin treatment inhibited neuronal apoptosis by downregulation of caspase-3. Given the importance of impaired autophagic influx in the pathology of ARHL and dysregulated autophagy is one of the core mechanisms underlying caspase-dependent neuronal apoptosis [48,49], we focused on the effects of neuritin treatments on the autophagic flux in the cochlea.

Our study showed that both endogenously and exogenously sourced neuritin administration could improve autophagic flux in the cochlea by increasing the expression of LC3B protein and decreasing the expression of p21 protein. In addition, 3-MA was used to block the synthesis of autophagic lysosomes to verify whether the promotion of autophagic flux mediates the protective effect of neuritin against cochlea injury. These results were consistent with those of previous studies, which found that neuritin could protect against neuronal injury by enhancing autophagic flux in the rat model of oxygen-glucose deprivation/reoxygenation (OGD/R)-induced neuronal injury [35] and subarachnoid hemorrhage (SAH) [34]. Autophagy plays a vital role in neuronal homeostasis and function. Moderate upregulation of autophagy has been shown to exert a protective function against HC dysfunction, SNG degeneration, and House Ear Institute-organ of Corti 1 (HEI-OC1) cell death in the cochlea, which are critical steps involved in the development of ARHL [50]. Given the central role of the autophagy signaling pathway in the pathology of ARHL, these results highlighted the potential therapeutic values of neuritin for this condition. Although the exact mechanisms by which neuritin regulates the autophagy signaling pathway have not been fully elucidated, there was evidence that neuritin could enhance autophagy by inhibiting the cGAS-stimulator of interferon genes (STING) pathway [34]. Moreover, neuritin was also demonstrated to accelerate Schwann cell differentiation via the PI3K/Akt/mTOR signaling pathway [51], which plays critical roles in both ARHL [52] and autophagy [53]. Neuritin treatment was also found to increase intracellular Ca^2+^ levels and the active Ca^2+^- calcineurin (CaN)-nuclear factor of activated T-cells (NFAT) c4 pathway in the neuron [54], which is critical for the Ca^2+^-dependent lysosomal enzyme activities and the formation of autophagosomes [55]. These signaling pathways may be potential mechanisms linking neuritin to autophagy. Further studies are warranted to identify the molecular mechanisms underlying the relationship between neuritin and autophagy.

In summary, our study reveals that overexpression of neuritin in SCs can restore hearing function and ameliorate the damages in HCs, nerve fibers, and SNGs in mice who experienced progressive ARHL. The underlying mechanisms may be associated with improving autophagy, reducing oxidative damage, and inhibiting hair cell apoptosis. These findings may contribute valuable insights into the prevention and treatment of ARHL. Moreover, as there was evidence that ARHL shares common cellular mechanisms with noise-induced hearing loss [56], it is worthy to further explore the preventive and therapeutic effects of neuritin on noise-induced hearing loss in the future.

## 4. Material and Methods

### 4.1. Ethical Assessment

Animal experiments were carried out strictly following the guidelines outlined in the National Institutes of Health (NIH) Guide for the Care and Use of Laboratory Animals. The study protocol was approved by the Animal Subjects Review Board of Hangzhou Normal University, with approval number 2024106. The surgical procedures were performed under anesthesia induced by sodium pentobarbital to ensure the well-being and comfort of the animals throughout the experiment. All methods are reported in accordance with ARRIVE guideline [57].

### 4.2. Generation of Sox2^CreER/+^ Neuritin^stop/+^ Mice and PCR Genotyping

Sox2^CreER/+^ Neuritin^stop/+^ mice were generated through a series of crosses involving CRISPR–Cas9 edited mice and CreER transgenic mice, as previously reported [32]. Briefly, using CRISPR–Cas9 genome editing technology, Neuritin^stop/+^ mice (F0) on a C57/BL6 background were created, following the method described by Yang et al. [58]. Then, these heterozygous mice were crossed to produce Nrn1^stop (-/-)^ mice (F1). The Sox2-CreER mouse line (no. 017593), sourced from the Jackson Laboratory, was used in conjunction with a gift of Sox2^CreER+^ (F1) mice from Professor Zhang Zunyi at Hangzhou Normal University, as described in Walters et al. [59]. To generate Sox2^CreER/+^ Neuritin^stop/+^ mice (F2), Nrn1^stop (-/-)^ mice were crossed with Sox2^CreER+^ mice. Subsequently, to produce the next generation (F3) of Sox2^CreER/+^ Neuritin^stop/+^ mice, the F2 mice were crossed back with Nrn1^stop (-/-)^ mice. The entire breeding strategy for these animals is outlined in detail in Figure S1A.

The PCR genotyping was conducted to ensure the successful generation of Sox2^CreER/+^ Neuritin^stop/+^ mice. The detailed protocol and specific primers for neuritin, Sox2, and the wild-type genes utilized in the genotyping process were described in a previous study [32]. In brief, genomic DNA was extracted from the tail tips of mice with a previously established protocol [60]. Then, a PCR reaction mixture (total of 20 μL) was prepared as follows: 1 µL of genomic DNA, 1.2 µL of primer mix (specific primers for Neuritin, Sox2, and the wild-type genes), 10 µL of 2× buffer, 4 µL of dNTPs, and 0.4 µL of KOD Fx enzyme (Toyobo Co., Ltd., Osaka, Japan). Water was added to make a total volume of 20 µL. The PCR reaction was carried out as follows: an initial denaturation step at 94 °C for 4 min, followed by 35 cycles of denaturation at 98 °C for 10 s, annealing at 60 °C for 30 s, and extension at 72 °C for 40 s. The genotyping results confirmed the successful generation of Sox2 ^CreER/+^ Nrn1^stop (+/-)^ transgenic mice, as shown in Figure S1B.

### 4.3. In Vivo Knock-In of Neuritin in ^Sox2+^ SCs in the Mouse Cochlea

Tamoxifen (Sigma-Aldrich, St Louis, MO, USA) was prepared in corn oil (Sigma-Aldrich, St Louis, MO, USA). It was administered to postnatal day (P)3 or P4 Sox2^CreER/+^ Nrn1^stop (+/-)^ mice (neuritin, experimental group) and Neuritin^stop/stop^ mice (Nrn1stop, controls) by intraperitoneal injections for three consecutive days [61]. The mice were sacrificed at four time points (12 weeks, 24 weeks, 36 weeks, and 48 weeks), and the expression levels of neuritin protein in the cochlea of Nrn1 KI mice and Nrn1stop mice were detected at different time points to confirm the constant overexpression of neuritin (Figure 1C). Moreover, the expression levels and cellular location of neuritin protein in the cochlea of wild-type mice (5-week-old) were detected by Western blot analysis and immunofluorescence staining to confirm the natural expression of neuritin protein in the cochlea of the mice (Figure 1A,B).

### 4.4. Preparation and Treatment of Cultured Cochlear Basilar Membranes (CBMs)

The cultured cochlear basilar membranes (CBMs) from neonatal mice were established by following a method previously described by Huang et al. [32]. Briefly, mouse pups were euthanized and decapitated, and the basilar membrane, including the organ of Corti and spiral ganglion neurons, was carefully isolated and placed on the surface of a collagen gel within a culture dish. Once the CBM adhered to the gel surface, serum-free medium was added, and the dish was incubated overnight. Subsequently, the cultured CBMs were divided into four groups, each containing six male mice: the control group received DMEM/F12 medium without any additives; the D-gal (L-D-gal) group was treated with DMEM/F12 medium containing 40 mg/mL of D-galactose; the Neuritin group was treated with DMEM/F12 medium containing both 40 mg/mL of D-galactose and 16 µg/mL of Neuritin; and the 3-MA group was treated with DMEM/F12 medium containing 40 mg/mL of D-galactose, 16 µg/mL of neuritin, and 2.0 nmol/mL of 3-MA. The dose of D-galactose (40 mg/mL) was selected based on our preliminary study, which indicated that 40 mg/mL of D-galactose treatment could induce significantly accelerated aging and elevated ROS levels in the CBMs (Figure 7A–C). After 48 h of culture, the CBMs were subjected to senescence-associated β-galactosidase (SA-β-GAL) staining, immunofluorescence staining, and Western blotting analysis.

### 4.5. Auditory Brainstem Response (ABR) Test

The auditory brainstem response (ABR) test of the mice was performed at time points of 12, 24, 36, and 48 weeks. Before the test, the mice were anesthetized via intraperitoneal injection of 0.01 g/mL pentobarbital sodium at a dose of 100 mg/kg body weight. The closed-field ABR thresholds of the mice were measured using a TDT System III workstation (Tucker-Davis Technologies, San Francisco, CA, USA), following a protocol previously described by Chen et al. [62]. The mice were placed on a thermostatic heating pad within a soundproof chamber, and three fine needle electrodes were inserted into specific locations on the mice: the cranial vertex, underneath the tested ear, and at the back near the tail. ABR tone pips of 8 kHz, 16 kHz, and 32 kHz were generated, and auditory thresholds were determined by gradually decreasing the sound intensities from 90 dB in 10 dB steps until the lowest sound intensity at which the first wave could be identified. The ABR data were analyzed using GraphPad Prism 9.0 software.

### 4.6. Immunostaining and Image Acquisition

To obtain cochlear tissues for immunofluorescence staining, mice were sacrificed, and the cochleae were dissected directly in cold Hank’s Balanced Salt Solution (HBSS) using sharp forceps. The dissected cochleae were then fixed in 4% paraformaldehyde at 4 °C for 24 h, while Corti organ tissues cultured in vitro were fixed in 4% paraformaldehyde at room temperature (RT) for 1 h. After fixation, the cochleae underwent decalcification with 0.5 M EDTA at RT for 3–7 days, depending on the age of the mouse.

After decalcification, the cochleae were dissected and incubated with a blocking solution containing 3% horse serum, 0.03% saponin, and 0.05% tween 20 in pH 7.4 phosphate-buffered saline (PBS) at RT for 2 h. The tissues were then incubated with primary antibodies diluted in an antibody dilution buffer (3% horse serum, 2 mg/mL bovine serum albumin, 0.05% tween 20 in pH 7.4 PBS) at 4 °C overnight. The primary antibodies used included anti-Myosin7a (myo7a; Proteus Bioscience, #25–6790; 1:1000 dilution), anti-NF200 (Santa Cruz Biotechnology, Santa Cruz, CA, USA, #17,320; 1:200 dilution), anti-LC3B (Cell Signaling Technology, #3868T, 1:100 dilution), and anti-p21 (Proteintech, #10355-1-AP; 1:100 dilution). Following primary antibody incubation, the tissues were washed and incubated with Alexa Fluor 488-conjugated secondary antibody (Invitrogen, Carlsbad, CA, USA) diluted 1:100 in antibody dilution buffer for 2 h at RT. Nuclei were stained with 20 μL of DAPI (2 μg/mL) for 10 min, and the sections were mounted with Fluorescence Mounting Medium (DAKO, Carpinteria, CA, USA, #S3203). Immunofluorescent images were captured using a Zeiss microscope (LSM 710, Tokyo, Japan) with consistent hardware settings for all images, allowing for direct comparisons between groups.

### 4.7. Cryosections

The cochleae of mice at different time points (12, 24, 36, and 48 weeks) were isolated, fixed in 4% paraformaldehyde, and decalcified with 0.5 M EDTA at 4 °C as mentioned above. Cochleae were then equilibrated with a series of ascending concentrations of sucrose (15–30%), initiated with 15% sucrose solution at 4 °C for 24 h, and then placed in 30% sucrose solution at 4 °C for 48 h. Tissues were then frozen in OCT, sectioned (20 μm thick) with a freezing microtome (Leica CM1950, Zeiss, Jena, Germany), and processed for immunofluorescence staining.

### 4.8. Western Blot Analysis

At least 10 cochleae from the mice or cultured cochlear basement membrane (CBM) samples were homogenized in 100 μL of ice-cold RIPA buffer (P0013B; Beyotime Institute of Biotechnology, Shanghai, China) containing a 1% protease inhibitor cocktail (Sigma, #P8340, St. Louis, MO, USA). The homogenate was centrifuged at 12,000× g for 10 min at 4 °C, and the resulting supernatant was collected. The protein concentration in the supernatant was determined using a BCA protein assay kit (Shenergy Biocolor Bioscience & Technology Company, Shanghai, China). For each well, 30–50 µg of total protein was loaded. The total protein was then subjected to electrophoresis on a 12% SDS-PAGE gel and transferred to PVDF membranes at 15 V for 30 min. The membranes were blocked with Tris-buffered saline (TBS) containing 5% fat-free skim milk for 2 h at room temperature. Following blocking, the membranes were incubated overnight at 4 °C with primary antibodies diluted in TBST (TBS + 0.1% Tween-20) containing 5% nonfat dried milk. The primary antibodies used were anti-neuritin (1:1000; ab64186, Abcam, London, UK), anti-LC3B (1:1000; #3868T, Cell Signaling Technology, Beverly, MA, USA), anti-p21 (1:1000; #10355-1-AP, Proteintech, Wuhan, China), anti-SQSTM1/p62 (1:1000; ab207305, Abcam, London, UK), anti-c-caspase-3 (1:1000; #9662, Cell Signaling Technology, Beverly, MA, USA), anti-caspase-3 (1:1000; #9664, Cell Signaling Technology, Beverly, MA, USA), and anti-β-actin (1:1000; ZSTA-09, ZSGB-BIO, Beijing, China). The next day, the membranes were washed and incubated with a secondary IgG-HRP antibody (1:2500; ZB-2301, ZSGB-BIO, Beijing, China) for 2 h at room temperature. Protein signals were detected using an ECL kit (Millipore, Billerica, MA, USA) in an automatic chemiluminescence/fluorescence image analysis system (5200 Multi, Tanon, Shanghai, China). Densitometric analysis of the images was performed using Adobe Photoshop CC 2015 software.

### 4.9. Senescence-Associated β-Galactosidase (SA-β-Gal) Activity Staining

The senescence assay for the cultured cochlear basement membranes (CBMs) was conducted by assessing the activity of senescence-associated β-galactosidase (SA-β-Gal). This was achieved by staining the CBMs according to the manufacturer’s instructions and previously established protocols [63]. The pH of the SA-β-Gal staining solution was maintained at 6.0 as specified. The procedure began by removing the cell culture medium and washing the CBMs once with phosphate-buffered saline (PBS). Subsequently, the CBMs were fixed for 15 min at room temperature, followed by overnight incubation at 37 °C, adhering strictly to the protocol provided with the senescence β-galactosidase kit (Beyotime Biotechnology, Shanghai, China). The development of a blue color served as an indication of SA-β-Gal activity within the CBMs. To observe and document this staining pattern, a light microscope was used, and images were captured immediately after the staining procedure using a camera (Nikon, Tokyo, Japan).

### 4.10. Detection of Reactive Oxygen Species (ROS) and Mitochondrial Fluorescent Probe Staining Analysis

DCFH-DA staining (D6883, Sigma Technologies) was employed to evaluate the cellular level of reactive oxygen species (ROS) in the cultured cochlear basement membranes (CBMs), following the manufacturer’s guidelines. Before staining, the CBMs were washed with serum-free Dulbecco’s Modified Eagle Medium (DMEM) and then incubated with a basal medium containing 10 μM DCFH-DA for 25 min at 37 °C in the dark. After three washes with phosphate-buffered saline (PBS), the fluorescent signal from the CBMs was captured using fluorescence microscopy or quantified using a FACS Calibur system (BD Biosciences, New York, NY, USA). Mito-SOX Red (Life Technologies, Carlsbad, CA, USA) was used with flow cytometry and fluorescence microscopy to detect mitochondrial ROS production specifically. Following drug administration, the cultured CBMs were incubated with 5 μM Mito-SOX Red for 15 min at 37 °C. Subsequently, the samples were examined under a laser scanning confocal microscope at a high-power field to visualize the fluorescent signal, thereby enabling the quantification of mitochondrial ROS production.

### 4.11. TUNEL Assay

To examine the presence of apoptotic cells in cultured cochlear basement membranes (CBMs), the one-step TUNEL (Terminal deoxynucleotidyl Transferase dUTP Nick End Labeling) kit (C1090, Beyotime Biotechnology, Shanghai, China) was utilized according to the manufacturer’s instructions. The procedure began by fixing the CBMs with 4% paraformaldehyde for 30 min and permeabilizing them with 1% TritonX-100 for 1 h. Next, the samples were equilibrated by covering them with 100 μL of equilibration buffer for 5 min at room temperature. After equilibration, the equilibration buffer was replaced with 50 μL of the terminal deoxynucleotidyl transferase reaction mix and incubated in the dark at 37 °C for 1 h. To visualize the actin cytoskeleton and provide additional context for the TUNEL staining, TRITC-phalloidin (Sigma) was used to co-label the tissues. Finally, the samples were observed under an Olympus BX63 microscope(Olympus, Tokyo, Japan).

### 4.12. Statistical Analysis

In our study, all experiments were meticulously conducted and independently repeated at least three times to ensure the reproducibility and reliability of our results. 

Data were presented as mean ± standard deviation (SD) and were analyzed with SPSS 22.0 and GraphPad Prism 5 software. Student’s *t*-test, one-way ANOVA followed by LSD post hoc test, and Bonferroni post hoc test were used for statistical analysis. All statistical values with a *p*-value less than 0.05 were considered statistically significant.

## Data Availability

The data that support the findings of this study are available from the corresponding author upon reasonable request.

## Supplementary Materials

The following supporting information can be downloaded at: https://www.mdpi.com/article/10.3390/ijms26083709/s1.
