# Supplementary material for: Conditional Overexpression of Neuritin in Supporting Cell Protects Cochlear Hair Cell and Delays Age-Related Hearing Loss by Enhancing Autophagy
Source: Int J Mol Sci. 2025 Apr 14;26(8):3709. doi: 10.3390/ijms26083709 (PMC12027747; doi:10.3390/ijms26083709)
Supplement: Supplementary file 1 [file ijms-26-03709-s001.zip › ijms-3543596-supplementary.pdf]

Conditional overexpression of neuritin in supporting cell protects cochlear hair cell and delays age-related hearing loss by enhancing autophagy

Shanshan Wang[#a], Shaowei Lv[#a], Junhao Hu[#a], Yunfan Shi[a], Yu Li[a], Jianyun Zhang[a], Xiaohua Tan[a], Rong Chen[a*], Yu Hong[a*]

[a] School of Public Health, Hangzhou Normal University, Hangzhou, Zhejiang, 311121, P.R. China

[#] These authors contributed equally to this work

* Correspondence authors.

E-mail addresses: rongchen1984@hznu.edu.cn (R. Chen); hongyu@hznu.edu.cn (Y. Hong).

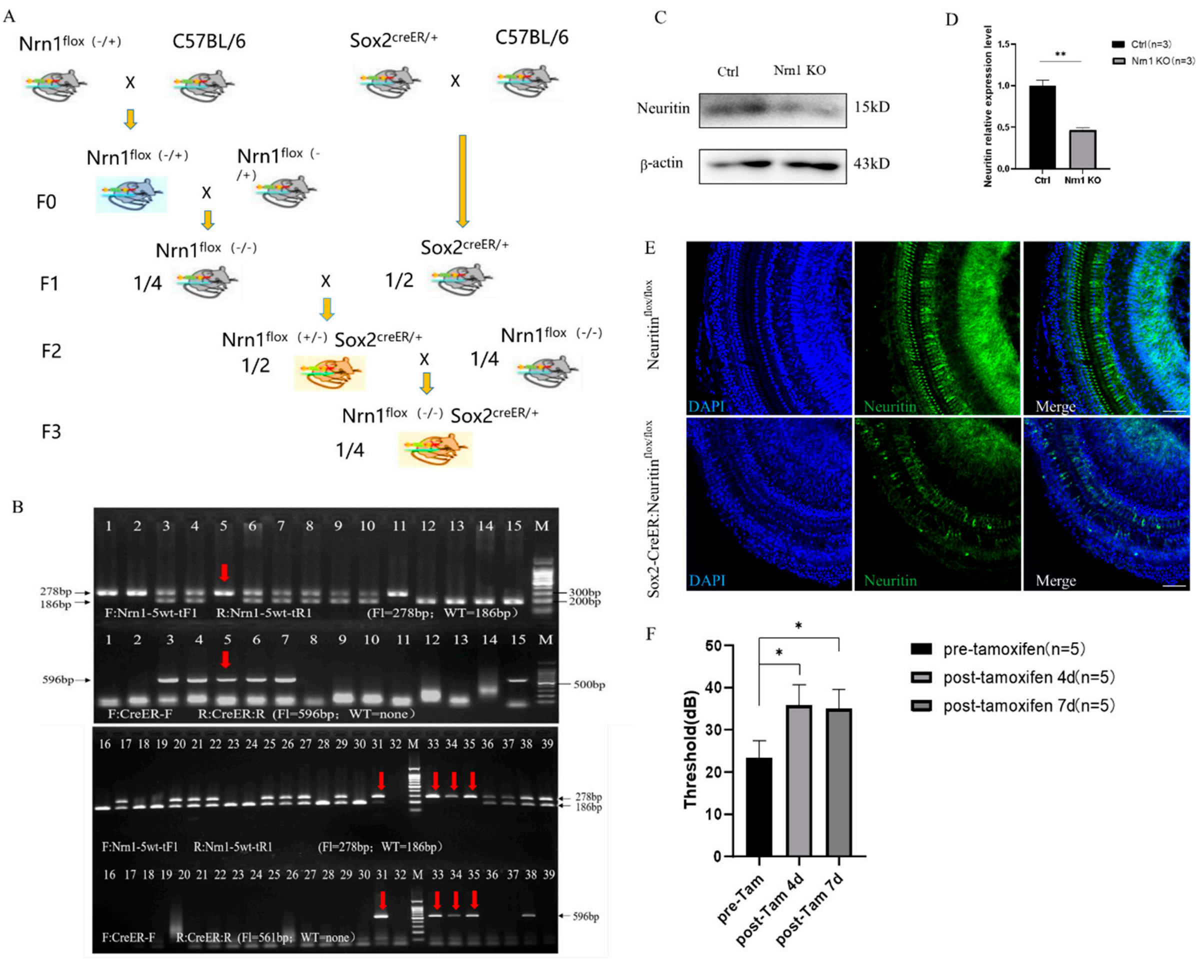

FIGURE S1 Reproductive strategy and genotyping of Neuritin conditional overexpression (Sox2$^{CreER/+}$Neuritin$^{stop/stop}$) mouse and the effects of conditional knockout of the Neuritin gene in wide-type mouse. (A) The reproductive strategy for mice with conditional overexpression of Neuritin (Sox2$^{CreER/+}$Neuritin$^{stop/stop}$). (B) Genotyping of Sox2$^{CreER/+}$Neuritin$^{stop/stop}$ mice. (C-D) Western blot and quantitative analysis of the expression levels of Neuritin protein in the cochlea of Neuritin *knock-out* (Nrn1 KO) and wild-type mice (Ctrl) (5-week-old). (E) The effects of conditional overexpression and *knock-out* of Neuritin gene in support cells (SCs) on the Neuritin protein levels in cochlea of mice. Green represents anti-EGFP labeling; purple represents DAPI-stained nucleus. Scale bar = 50 μm. (F)The hearing threshold elicited by click auditory brainstem response (ABR) test in Neuritin *knock-out* (Nrn1

KO) mice at different time points after tamoxifen treatment. Statistical analyses were performed by one-way ANOVA followed by LSD post-hoc test. *, $p < 0.05$; **, $p < 0.01$; ***, $p < 0.001$.
